# Association Between Neutrophil‐to‐Lymphocyte Ratio and Glycemic Control in Type 2 Diabetes Mellitus: An Updated Systematic Review and Meta‐Analysis

**DOI:** 10.1002/hsr2.71784

**Published:** 2026-02-01

**Authors:** Maryam Mohammadi, Parsa Panahiyan, Faizan Bashir, Shayesteh Haghighi, Sara Ahmadi, Ensiyeh Olama, Yasaman Ghodsi Boushehri, Elnaz Olama, Alaleh Alizadeh, Zahra Sadat Hoseini Nasab, Mahdyieh Naziri, Niloofar Deravi, Neda Fakhrghasemi, Danyal Yarahmadi

**Affiliations:** ^1^ Islamic Azad University of Najafabad Najafabad Iran; ^2^ Faculty of Medicine Gaziantep University Gaziantep Turkey; ^3^ School of Medicine Shiraz University of Medical Sciences Shiraz Iran; ^4^ Department of Nursing, School of Nursing and Midwifery Ahvaz Jundishapur University of Medical Sciences Ahvaz Iran; ^5^ School of Medicine Shahid Beheshti University of Medical Sciences Tehran Iran; ^6^ Student Research Committee, School of Medicine Georgian National University SEU Tbilisi Georgia; ^7^ Shiraz University of Medical Sciences Shiraz Iran; ^8^ Faculty of Medicine Georgian National University SEU Tbilisi Georgia; ^9^ Faculty of Medicine Mashhad Branch, Islamic Azad University Mashhad Iran; ^10^ School of Medicine Mashhad University of Medical Sciences Mashhad Iran; ^11^ Student Research Committee, Department of Biostatistics, School of Public Health Iran University of Medical Sciences Tehran Iran; ^12^ Student Research Committee, School of Medicine Shahid Beheshti University of Medical Sciences Tehran Iran; ^13^ Allergy Research Center Mashhad University of Medical Sciences Mashhad Iran; ^14^ Student Research Committee, School of Medicine Iran University of Medical Sciences Tehran Iran; ^15^ Razi Herbal Medicines Research Center Lorestan University of Medical Sciences Khorramabad Iran

**Keywords:** glycemic control, hemoglobin A1c (HbA1c), inflammation, Neutrophil‐to‐Lymphocyte Ratio (NLR), systemic inflammation, Type 2 Diabetes Mellitus (T2DM)

## Abstract

**Background:**

Type 2 diabetes mellitus (T2DM) is increasingly seen as a chronic inflammatory state, with the neutrophil‐to‐lymphocyte ratio (NLR) studied as a new biomarker of systemic inflammation. Evidence links NLR to glycemic control, but its consistency and strength vary. This review summarizes data on NLR's relationship with glycemic indicators, such as hemoglobin A1c (HbA1c), across different T2DM glycemic states.

**Methods:**

A systematic literature search was carried out in PubMed/MEDLINE, Scopus, Web of Science, and Embase through November 2025 to find studies that reported both NLR and glycemic control indicators in adults with T2DM. Data were systematically extracted based on predefined criteria. Standardized mean differences (SMDs) with 95% confidence intervals (CIs) were combined using random‐effects models. Heterogeneity was measured using the I² statistic, and robustness was assessed using sensitivity analyses. Publication bias was investigated using Begg's and Egger's tests, trim‐and‐fill analysis, and funnel plot examination. All statistical analyses were conducted using STATA 18 (StataCorp, College Station, TX, USA).

**Results:**

The meta‐analysis consisted of 27 studies (37 comparisons) with a total of 48,714 individuals from 11 nations. NLR demonstrated a stepwise gradient with deteriorating glycemic control: poorly controlled T2DM (HbA1c >  7%) had higher NLR than well‐controlled T2DM (HbA1c ≤  7%) (*p* <  0.001), prediabetes (HbA1c 5.7–6.4%) (*p* <  0.001), and normoglycemia (HbA1c ≤  5.7%) (*p* =  0.037).

**Conclusions:**

NLR rises progressively with deteriorating glycemic control and is markedly raised in poorly controlled T2DM. These observations endorse NLR as an inexpensive, widely available inflammatory biomarker that can augment conventional glycemic measures in the clinical setting.

**Abbreviations**
BMI: body mass index
CI: confidence interval
CRP: C‐reactive protein
FBG: fasting blood glucose
GLP‐1: glucagon‐like peptide‐1
HbA1c: Hemoglobin A1c
HR: hazard ratio
IL‐6: Interleukin‐6
IPD: individual participant data
JBI: Joanna Briggs Institute
MeSH: Medical Subject Headings
NLR: Neutrophil‐to‐Lymphocyte Ratio
PRISMA: Preferred reporting items for systematic reviews and meta‐analyses
PROSPERO: international prospective register of systematic reviews
REML: restricted maximum‐likelihood
SD: standard deviation
SGLT2: sodium‐glucose cotransporter‐2
SMD: standardized mean difference
T2DM: type 2 diabetes mellitus
TNF‐α: tumor necrosis factor‐alpha
WBC: white blood cell

## 1 Background

Type 2 diabetes mellitus (T2DM) is a worldwide health challenge, with its prevalence rising at an alarming rate across the world and especially in Asia. This has been mainly due to urbanization, unhealthy diets, and increasing physical inactivity. People of Asian origin are not only more prone to developing T2DM at an early age and lower body mass index (BMI), but this susceptibility has also been associated with excessive carbohydrate intake, smoking, and alcohol consumption. Although genetic research has shed light on certain risk factors, lifestyle changes continue to be the foundation of T2DM prevention [1].

It has been universally accepted that optimal glycemic control is vital in patients with T2DM. Ample epidemiological data indicate that good control can prevent or at least postpone the development and progression of the myriad vascular complications of the disease [2].

The raised circulating leukocyte count is not only a marker of acute infection but also plays a central role in the inflammatory processes that mediate the initiation, progression, and complications of atherosclerosis. This inflammatory process can ultimately lead to rupture of atherosclerotic plaques and cardiovascular events. Numerous studies have consistently demonstrated that elevated leukocyte counts are consistent markers of systemic inflammation, providing important diagnostic and prognostic information in patients with angina, myocardial infarction, stroke, peripheral vascular disease, and microvascular and macrovascular complications of diabetes [3, 4]. Neutrophils, the most common leukocytes in the blood, respond quickly to inflammatory stimuli, leading to increased circulation. Interleukin levels are also increased in inflammatory conditions, and they induce lymphopenia and neutrophilia, causing a high NLR [5, 6]. A high neutrophil count reflects an active, non‐specific, and destructive inflammatory process, while a low lymphocyte count indicates impaired immune regulation and a weakened immune response [7].

Growing interest is being put on the NLR as an easily obtained yet valuable marker of inflammation in cardiac and non‐cardiac pathologies. The NLR has been deemed a more predictive, diagnostic, and discriminative biomarker compared to the total white blood cell (WBC) count. In addition, its predictive potential equals that of other traditional inflammatory markers, including C‐reactive protein (CRP), tumor necrosis factor (TNF‐α), and interleukin‐6 (IL‐6), for detecting subclinical inflammation and endothelial dysfunction, as evidenced in several clinical studies [8, 9].

The relationship between diabetes mellitus (DM) and the NLR has been a subject of active investigation recently [10]. The main aim of this systematic review and meta‐analysis is to investigate the possible usefulness of NLR as an indicator of glycemic control in T2DM patients.

## 2 Methods

### 2.1 Study Design and Registration

We performed a systematic review and meta‐analysis to examine the association between the NLR and glycemic control in adults with T2DM. Methods followed the Preferred Reporting Items for Systematic Reviews and Meta‐Analyses (PRISMA) 2020 checklist [11].

### 2.2 Literature Search

A systematic literature search was conducted in PubMed/MEDLINE, Scopus, Web of Science and Embase from their inception to November 2025. MeSH and free‐text terms for neutrophil, lymphocyte, NLR, HbA1c, fasting glucose, and type 2 diabetes were used, combined with Boolean operators and customized for each database. No language or date limits were applied to the search. The reference lists of all qualifying papers and of previous systematic reviews were also manually searched. Two independent reviewers screened titles and abstracts; any discrepancies were resolved through discussion (Table 1).

**Table 1:** Search strategies for PubMed/MEDLINE, Embase, Scopus databases and Web of Science.

Search engine	Search strategy	Search date	Search results
PubMed/MEDLINE	(“Neutrophil‐to‐Lymphocyte Ratio”[Title/Abstract] OR “NLR”[Title/Abstract] OR “Neutrophil Lymphocyte Ratio”[Title/Abstract]) AND (“Glycemic”[Title/Abstract] OR “HbA1c”[Title/Abstract] OR “Glycated hemoglobin”[Title/Abstract] OR “Hemoglobin”[Title/Abstract] OR “Glycemia”[Title/Abstract] OR “Glycemic Control”[Title/Abstract]) AND (“Diabetes Mellitus”[MeSH Terms] OR “T2DM”[Title/Abstract] OR “diabetes mellitus type 2”[Title/Abstract] OR “Glucos”[Title/Abstract] OR “Fasting blood sugar”[Title/Abstract] OR “postprandial blood sugar”[Title/Abstract])	2025/11/26	140
Embase	(“Neutrophil‐to‐Lymphocyte Ratio”:ti, ab OR “NLR”:ti, ab OR “Neutrophil Lymphocyte Ratio”:ti, ab) AND (“Glycemic”:ti, ab OR “HbA1c”:ti, ab OR “Glycated hemoglobin”:ti, ab OR “Hemoglobin”:ti, ab OR “Glycemia”:ti, ab OR “Glycemic Control”:ti, ab) AND (‘diabetes mellitus type 2’/exp OR “T2DM”:ti, ab OR “diabetes mellitus type 2”:ti, ab OR “Glucose”:ti, ab OR “Fasting blood sugar”:ti, ab OR “Postprandial blood sugar”:ti, ab)	2025/11/26	358
Scopus	TITLE‐ABS‐KEY ((“Neutrophil‐to‐Lymphocyte Ratio” OR “NLR” OR “Neutrophil Lymphocyte Ratio”) AND (“Glycemic” OR “HbA1c” OR “Glycated hemoglobin” OR “Hemoglobin” OR “Glycemia” OR “Glycemic Control”) AND (“Diabetes Mellitus” OR “T2DM” OR “diabetes mellitus type 2” OR “Glucose” OR “Fasting blood sugar” OR “postprandial blood sugar”))	2025/11/26	1977
Web of Science	(TI = (“Neutrophil‐to‐Lymphocyte Ratio” OR “NLR” OR “Neutrophil Lymphocyte Ratio”) OR AB = (“Neutrophil‐to‐Lymphocyte Ratio” OR “NLR” OR “Neutrophil Lymphocyte Ratio”)) AND (TI = (“Glycemic” OR “HbA1c” OR “Glycated hemoglobin” OR “Hemoglobin” OR “Glycemia” OR “Glycemic Control”) OR AB = (“Glycemic” OR “HbA1c” OR “Glycated hemoglobin” OR “Hemoglobin” OR “Glycemia” OR “Glycemic Control”)) AND (TI = (“Diabetes Mellitus” OR “T2DM” OR “diabetes mellitus type 2” OR “Glucose” OR “Fasting blood sugar” OR “postprandial blood sugar”) OR AB = (“Diabetes Mellitus” OR “T2DM” OR “diabetes mellitus type 2” OR “Glucose” OR “Fasting blood sugar” OR “postprandial blood sugar”))	2025/11/26	233

### 2.3 Eligibility Criteria

Studies were eligible for inclusion if they:

- Used an observational study design (i.e., cross‐sectional, cohort, or case‐control).
- Enrolled adult patients with a clinical diagnosis of T2DM;
- Reported both NLR and at least one marker of glycemic control (HbA1c or fasting blood glucose);
- Supplied enough data to calculate an effect size.

Exclusion criteria were animal studies, randomized controlled trials involving active interventions, conference abstracts without complete data, and articles without extractable NLR data.

### 2.4 Data Extraction

Two reviewers separately extracted data from each included study, including the first author's name, year of publication, country, study design, and sample size. The primary outcome of our meta‐analysis was the standardized mean difference (SMD) in NLR across glycemic control groups. When the mean and standard deviation (SD) were reported in studies, they were used directly. For studies that reported the median, minimum, and maximum, we estimated the mean and SD using the method reported by Hozo et al., 2005.

### 2.5 Risk of Bias Assessment

The risk of bias and methodological quality of each study were critically assessed independently by two reviewers using the relevant Joanna Briggs Institute (JBI) critical appraisal checklists for cross‐sectional, case‐control, and cohort studies. A quality score was calculated for each study as the percentage of ‘Yes’ responses to the relevant checklist questions. The studies were then rated ‘high quality’ if their score was 70% or higher, ‘moderate quality’ if their score was 50–69%, and ‘low quality’ if their score was 49% or lower. Any differences in the appraisal were settled by discussion with a third reviewer. Table 2 presents the outcomes of this quality appraisal, including the scores per study.

**Table 2:** Summary of Study Characteristics.

First Author	Year	Country	Study design	n (Good/Poor)	Age (Good/Poor) mean (SD)	Female% (Good/Poor)	BMI (Good/Poor) mean (SD)	Duration (yrs) (Good/Poor)	NLR (Good/Poor) mean (SD)	HbA₁c (Good/Poor) mean (SD)	JBI Score
N. Taban	2025	Iran	case‐control	300/300	55.90 (8.10)/56.20 (8.40)	55%/51.61%	24.80 (3.60)/29.50 (4.80)	NA (NA)/NA (NA)	1.77 (1.13)/3.12 (1.66)	4.70 (0.80)/9.80 (2.80)	8/10
B. Yazar	2025	Turkey	case‐control	50/50	46.00 (19.00)/63.00 (12.50)	NA/NA	NA (NA)/NA (NA)	NA (NA)/NA (NA)	1.88 (0.57)/2.27 (1.48)	4.80 (0.80)/8.40 (1.45)	7/10
C. Mertoglu	2016	Turkey	cross‐sectional	9/34	NA (NA)/NA (NA)	NA/NA	NA (NA)/NA (NA)	NA (NA)/NA (NA)	1.58 (0.78)/2.07 (0.95)	5.05 (3.15)/7.86 (3.34)	6/8
C. Mertoglu	2016	Turkey	cross‐sectional	42/34	NA (NA)/NA (NA)	NA/NA	NA (NA)/NA (NA)	NA (NA)/NA (NA)	1.37 (0.69)/2.07 (0.95)	5.16 (2.19)/7.86 (3.34)	6/8
Sharmila Dudani	2021	India	cross‐sectional	69/60	50.40 (10.64)/49.70 (8.87)	46%/48%	NA (NA)/NA (NA)	NA (NA)/NA (NA)	2.02 (0.71)/1.65 (0.56)	5.19 (0.18)/7.12 (1.77)	7/8
C. Mertoglu	2016	Turkey	cross‐sectional	25/34	NA (NA)/NA (NA)	NA/NA	NA (NA)/NA (NA)	NA (NA)/NA (NA)	1.60 (0.85)/2.07 (0.95)	5.26 (2.57)/7.86 (3.34)	6/8
I.J. Martins	2024	Brazil	cross‐sectional	230/229	NA (NA)/NA (NA)	57%/62%	NA (NA)/NA (NA)	NA (NA)/NA (NA)	1.80 (0.85)/1.88 (0.84)	5.30 (0.52)/8.70 (2.92)	6/8
K. Essawi	2023	Saudi Arabia	case‐control	175/250	32.40 (7.20)/53.00 (12.00)	NA/NA	27.60 (6.80)/29.70 (11.70)	12.00 (7.40)/NA (NA)	1.20 (0.60)/1.40 (0.70)	5.30 (0.70)/9.20 (2.20)	8/10
B. Ünal	2025	Turkey	cross‐sectional	500/498	39.70 (14.14)/56.94 (11.18)	69.2%/61.4%	NA (NA)/NA (NA)	NA (NA)/NA (NA)	1.93 (0.86)/2.08 (1.02)	5.43 (0.35)/7.56 (1.73)	7/8
Mazhar Hussain	2017	Pakistan	cross‐sectional	110/110	62.60 (13.00)/64.60 (15.90)	52.7%/56.4%	26.50 (2.80)/27.20 (3.20)	NA (NA)/NA (NA)	2.00 (2.70)/2.10 (3.00)	5.50 (NA)/8.50 (NA)	7/8
P.P. Wu	2019	China	cross‐sectional	100/100	57.25 (2.68)/58.19 (2.81)	47%/40%	NA (NA)/NA (NA)	NA (NA)/NA (NA)	1.80 (0.17)/1.81 (0.19)	5.53 (0.084)/8.53 (0.50)	7/8
Y. Turan	2019	Turkey	cross‐sectional	40/92	48.68 (6.99)/49.53 (4.10)	67.5%/61.96%	30.25 (6.10)/33.94 (6.35)	NA (NA)/NA (NA)	1.72 (0.61)/2.32 (1.10)	5.62 (0.39)/9.16 (2.16)	7/8
A.M. Mohammad	2024	India	case‐control	132/160	61.50 (14.44)/65.80 (10.74)	NA/55.63%	NA (NA)/NA (NA)	NA (NA)/NA (NA)	4.09 (8.85)/4.18 (4.15)	5.66 (0.52)/8.16 (1.60)	7/10
M. Elsanan	2023	Egypt	cross sectional	30/30	NA (NA)/NA (NA)	NA/NA	NA (NA)/NA (NA)	NA (NA)/NA (NA)	2.00 (0.60)/2.30 (1.10)	5.80 (0.20)/9.30 (0.70)	6/8
M. Elsanan	2024	Egypt	cross sectional	30/30	56.77 (7.81)/56.50 (8.96)	53.3%/46.7%	NA (NA)/NA (NA)	NA (NA)/NA (NA)	2.00 (0.60)/2.30 (1.10)	5.80 (0.20)/9.30 (0.70)	7/8
K. SIGAMANI	2025	India	cross‐sectional	62/81	45.20 (13.75)/52.60 (10.43)	50%/45.7%	NA (NA)/NA (NA)	NA (NA)/NA (NA)	2.61 (2.88)/3.52 (2.39)	5.85 (NA)/9.95 (NA)	7/8
B. Yazar	2025	Turkey	case‐control	36/50	65.00 (18.50)/63.00 (12.50)	NA/NA	NA (NA)/NA (NA)	NA (NA)/NA (NA)	1.87 (0.91)/2.27 (1.48)	5.90 (0.30)/8.40 (1.45)	6/8
I.J. Martins	2024	Brazil	cross‐sectional	140/229	NA (NA)/NA (NA)	69.3%/62.4%	NA (NA)/NA (NA)	NA (NA)/NA (NA)	1.62 (0.83)/1.88 (0.84)	5.90 (0.20)/8.70 (2.92)	7/8
M. Lou	2015	China	case‐control	130/103	64.39 (6.17)/63.55 (4.60)	77.7%/75.7%	23.76 (3.53)/23.65 (3.66)	NA (NA)/NA (NA)	1.42 (0.30)/1.71 (0.50)	5.99 (1.49)/7.25 (1.84)	9/10
S. Abhijt	2014	India	cross sectional	63/286	43.00 (8.00)/47.00 (8.00)	NA/NA	25.00 (3.10)/25.60 (4.30)	NA (NA)/NA (NA)	1.82 (0.60)/2.20 (1.10)	6.10 (0.60)/8.50 (2.20)	7/8
A. Kesharwani	2025	India	cross‐sectional	109/108	NA (NA)/NA (NA)	NA/NA	NA (NA)/NA (NA)	NA (NA)/NA (NA)	2.40 (1.24)/2.56 (2.20)	6.11 (0.47)/9.48 (2.21)	6/8
K. Aygun	2024	Turkey	case‐control	327/163	59.75 (11.85)/60.75 (10.86)	NA/NA	NA (NA)/NA (NA)	NA (NA)/NA (NA)	1.96 (0.61)/2.26 (0.81)	6.15 (0.067)/9.58 (1.92)	8/10
E. Palella	2020	Italy	case‐control	58/75	64.63 (10.90)/66.43 (10.40)	51.7%/45.3%	NA (NA)/NA (NA)	NA (NA)/NA (NA)	1.90 (0.80)/2.28 (0.97)	6.20 (0.40)/8.50 (1.60)	8/10
Fatih Sefil	2014	Turkey	Retrospective cohort	34/37	55.90 (11.60)/57.70 (9.80)	52.9%/54.1%	34.10 (5.50)/30.60 (5.00)	7.00 (6.30)/6.50 (5.90)	1.45 (0.56)/1.97 (0.56)	6.30 (0.40)/8.90 (1.10)	10/11
T. Assulyn	2020	Israel	Cohort	53/57	64.00 (11.00)/61.00 (10.00)	54.7%/52.6%	28.81 (5.64)/29.06 (4.73)	10.00 (6.00)/14.00 (8.00)	1.90 (0.65)/2.06 (0.83)	6.30 (0.46)/8.43 (1.08)	11/11
K. Essawi	2023	Saudi Arabia	case‐control	28/92	51.50 (12.60)/53.90 (11.30)	NA/NA	27.30 (3.90)/31.60 (16.60)	9.80 (6.10)/10.80 (6.50)	1.30 (0.60)/1.40 (0.70)	6.50 (0.40)/8.00 (0.60)	9/10
I.J. Martins	2024	Brazil	cross‐sectional	22/229	NA (NA)/NA (NA)	59.1%/62.4%	NA (NA)/NA (NA)	NA (NA)/NA (NA)	1.96 (0.61)/1.88 (0.84)	6.70 (0.10)/8.70 (2.92)	6/8
Abdullah	2025	India	case‐control	80/80	55.24 (10.83)/57.36 (8.35)	52.5%/42.5%	NA (NA)/NA (NA)	5.45 (3.61)/11.60 (3.25)	1.91 (0.61)/2.15 (0.50)	6.80 (0.40)/8.13 (0.70)	8/10
M. Cojic	2024	Montenegro	cross sectional	92/48	59.52 (13.56)/66.77 (7.56)	50%/52.1%	27.66 (4.91)/29.24 (3.59)	7.30 (5.04)/7.28 (4.94)	1.68 (1.05)/1.49 (0.76)	6.85 (1.47)/8.17 (1.88)	8/8
M. Cojic	2024	Montenegro	cross sectional	92/211	59.52 (13.56)/67.23 (10.00)	50%/51.2%	27.66 (4.91)/28.22 (4.70)	7.30 (5.04)/9.08 (4.84)	1.68 (1.05)/1.72 (0.80)	6.85 (1.47)/7.23 (1.57)	8/8
M. Cojic	2024	Montenegro	cross sectional	92/73	59.52 (13.56)/71.23 (9.04)	50%/58.9%	27.66 (4.91)/28.97 (4.56)	7.30 (5.04)/10.11 (4.50)	1.68 (1.05)/2.31 (1.41)	6.85 (1.47)/7.35 (1.79)	7/8
A.A. Alnabi	2020	Syria	cross‐sectional	120/120	NA (NA)/NA (NA)	NA/NA	NA (NA)/NA (NA)	NA (NA)/NA (NA)	1.50 (0.20)/2.98 (0.60)	NA (NA)/NA (NA)	6/8
B.B. Mendes	2018	Brazil	cross‐sectional	139/139	66.25 (7.75)/61.00 (8.67)	4.3%/24.5%	NA (NA)/NA (NA)	NA (NA)/NA (NA)	3.45 (2.00)/2.60 (1.93)	NA (NA)/NA (NA)	7/8
N. Najeeb	2019	India	cross‐sectional	110/220	62.60 (13.00)/64.60 (15.90)	52.7%/56.4%	26.50 (2.80)/27.20 (3.20)	NA (NA)/NA (NA)	2.00 (0.50)/3.50 (2.25)	NA (NA)/NA (NA)	7/8
Nurahami	2021	Indonesia	cross‐sectional	20/36	NA (NA)/NA (NA)	NA/NA	NA (NA)/NA (NA)	NA (NA)/NA (NA)	1.52 (0.50)/1.90 (0.84)	NA (NA)/NA (NA)	6/8
Sharmila Dudani	2021	India	cross‐sectional	20/40	39.70 (14.14)/56.94 (11.18)	NA/NA	NA (NA)/NA (NA)	NA (NA)/NA (NA)	2.06 (0.77)/1.94 (0.61)	NA (NA)/NA (NA)	6/8
T.T. Duman	2019	Turkey	cross sectional	33/77	39.20 (12.40)/58.60 (10.90)	33.3%/48.1%	25.40 (4.30)/30.90 (5.50)	NA (NA)/NA (NA)	1.50 (0.90)/2.44 (1.90)	NA (NA)/NA (NA)	7/8

### 2.6 Statistical Analysis

For each study, standardized mean differences (SMDs) with 95% confidence intervals (CIs) were calculated for the NLR difference between groups. Analyses were stratified into three contrasts:

- Poorly controlled diabetes (HbA1c >  7%) vs well‐controlled diabetes (HbA1c ≤  7%).
- Poorly controlled diabetes vs prediabetes (HbA1c 5.7–6.4%).
- Poorly controlled diabetes vs normoglycemia (HbA1c <  5.7%).

Random‐effects models were fitted with the restricted maximum‐likelihood (REML) estimator; the I² statistic quantified between‐study heterogeneity [12], with *I*² > 50% denoting substantial heterogeneity [13]. Sensitivity analyses were performed by sequentially omitting each study. Publication bias was evaluated by Begg's rank‐correlation test, Egger's regression asymmetry test, trim‐and‐fill analysis, and inspection of funnel‐plot symmetry [14]. We performed subgroup analyses stratified by geographic region (Africa, Asia, Europe, North America) to explore whether between‐study heterogeneity differed across settings. All computations were conducted using STATA 18 (StataCorp, College Station, TX, USA).

## 3 Results

### 3.1 Study Selection and Characteristics

**Figure 1:**
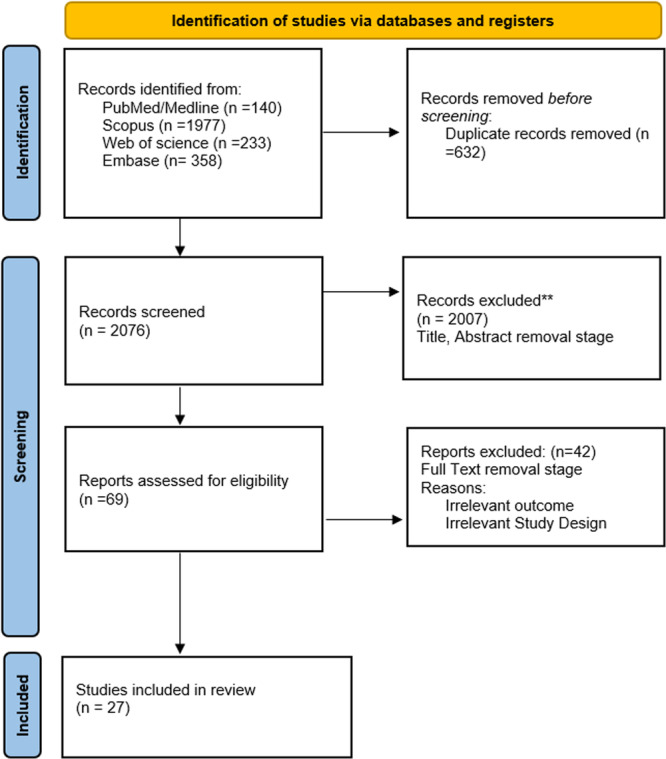
PRISMA flow diagram illustrating the study selection process.

We included 27 qualifying studies with 37 comparative analyses in this meta‐analysis, representing data on 48,714 adults with T2DM from 11 countries. Figure 1 (PRISMA Flow Diagram) describes the process for study selection. Most studies were cross‐sectional, while the remaining few were retrospective cohort analyses.

The demographic characteristics of participants reported a weighted average age of 57 years, a female representation of 49%, and a mean BMI of 28.6 kg/m², placing them in the overweight/obese range. Participants with poorly controlled glycemic status showed considerably higher concentrations of HbA1c (8.4% mean), FBG, and NLR levels compared to individuals with controlled diabetes (mean HbA1c: 6.3%) (Table 2).

### 3.2 Meta‐Analysis of NLR Differences by Glycemic Control

**Figure 2:**
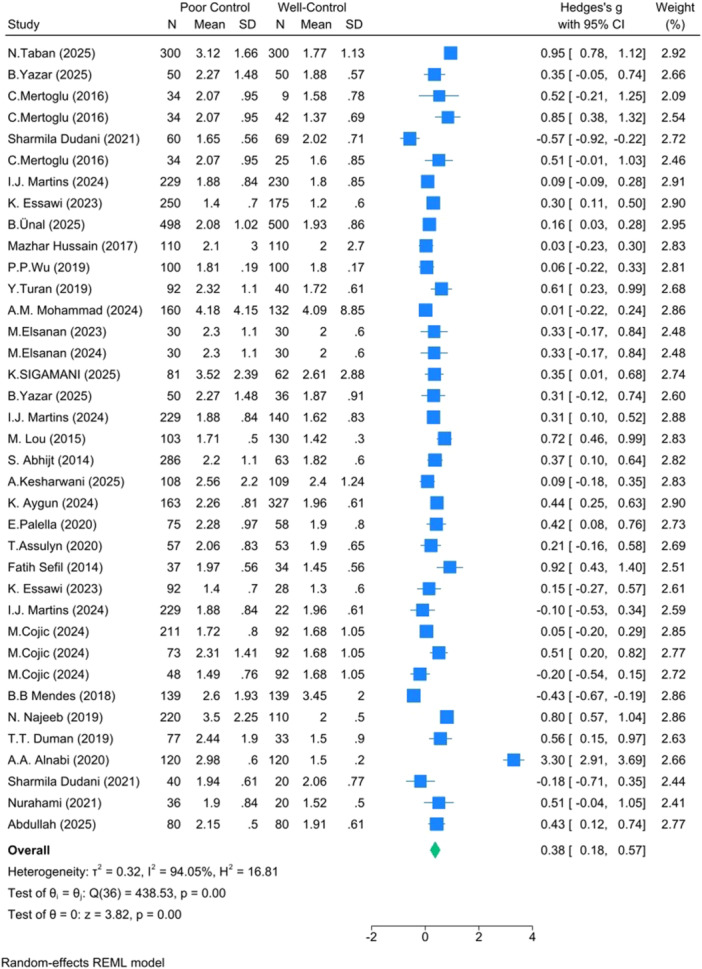
Forest plot for poor versus well‐controlled T2DM.

**Figure 3:**
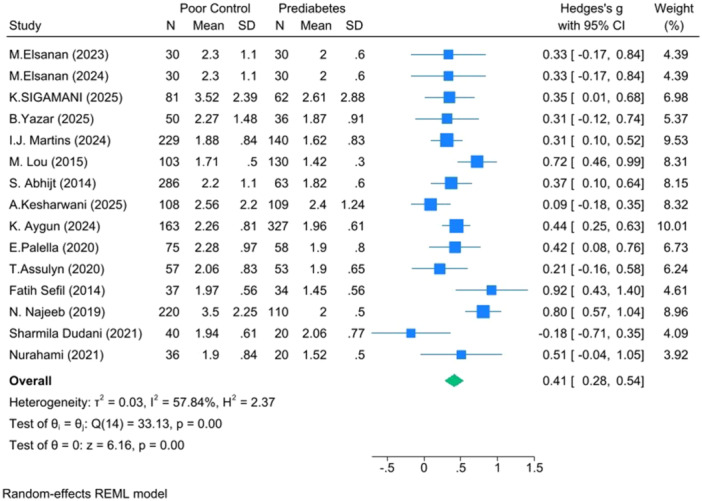
Forest plot for poor versus prediabetes.

**Figure 4:**
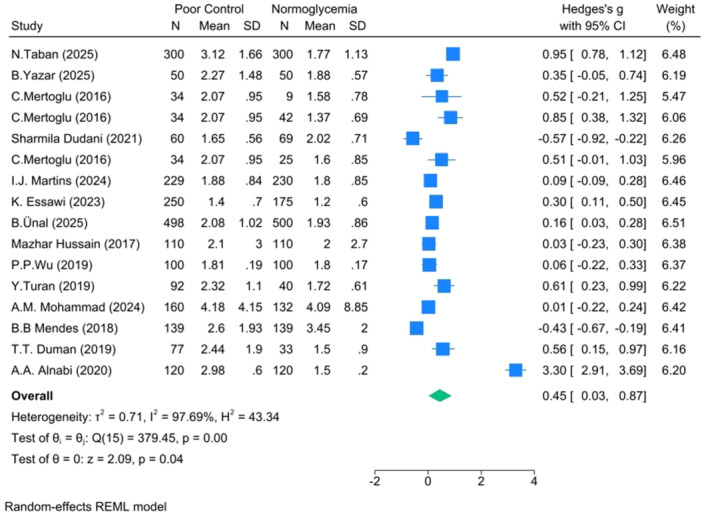
Forest plot for poor versus normoglycemia.

Meta‐analysis comparisons indicated a stepwise gradient in NLR with worsening glycemic status:

- **Poor versus Well‐Controlled T2DM** (*k* =  37): Hedges's *g* =  0.38, 95% CI: 0.18–0.57, *p* <  0.001, I² = 94.05% (Figure 2).
- **Poor versus Prediabetes** (*k* =  15): Hedges's g =  0.41, 95% CI: 0.28–0.54, *p* <  0.001, I² = 57.84% (Figure 3).
- **Poor versus Normoglycemia** (*k* =  16): Hedges's *g* =  0.45, 95% CI: 0.03–0.87, *p* =  0.037, I² = 97.69% (Figure 4).

These results are comprehensively tabulated in Table 3, demonstrating a definite gradient of increasing NLR with worsening glycemic status, with the Poor versus Prediabetes comparison showing the most consistent and homogeneous effect across studies.

**Table 3:** Summary of meta‐analysis results for NLR differences.

Comparison	Total Participants	Hedges's *g*	95% CI	*z*‐value	*p* value	I² (%)	τ² (Tau‐squared)	H² (H‐squared)	Q‐statistic	Q *p* value
Poor vs Well‐controlled T2DM	48,714	0.38	0.18–0.57	3.82	< 0.001	94.05	0.32	16.81	438.53	< 0.001
Poor vs Prediabetes	8432	0.41	0.28–0.54	6.16	< 0.001	57.84	0.03	2.37	33.13	< 0.001
Poor vs Normoglycemia	6847	0.45	0.03–0.87	2.09	0.037	97.69	0.71	43.34	379.45	< 0.001

### 3.3 Sensitivity and Heterogeneity Analyses

There was considerable heterogeneity in the Poor versus Well‐controlled (*I*² = 94.05%) and Poor versus Normoglycemia (*I*² = 97.69%) comparisons, but moderate heterogeneity in the Poor versus Prediabetes comparison (*I*² = *57.84%*). The robustness of the pooled effect sizes was evaluated via leave‐one‐out sensitivity analyses:

- **Poor versus Well‐controlled**: The effect remained stable (*g* ≈ 0.36–0.40) and statistically significant across all iterations, confirming high robustness.
- **Poor versus Prediabetes**: The effect remained highly stable (g ≈ 0.37–0.44) with no single study altering the significance, indicating excellent consistency.
- **Poor versus Normoglycemia**: The effect was somewhat sensitive; removal of certain studies (e.g., N. Taban 2025, C. Mertoglu 2016) rendered the result marginally non‐significant (*p* ≈  0.06–0.07), though the effect remained positive in all iterations, suggesting moderate robustness.

**Figure 5:**
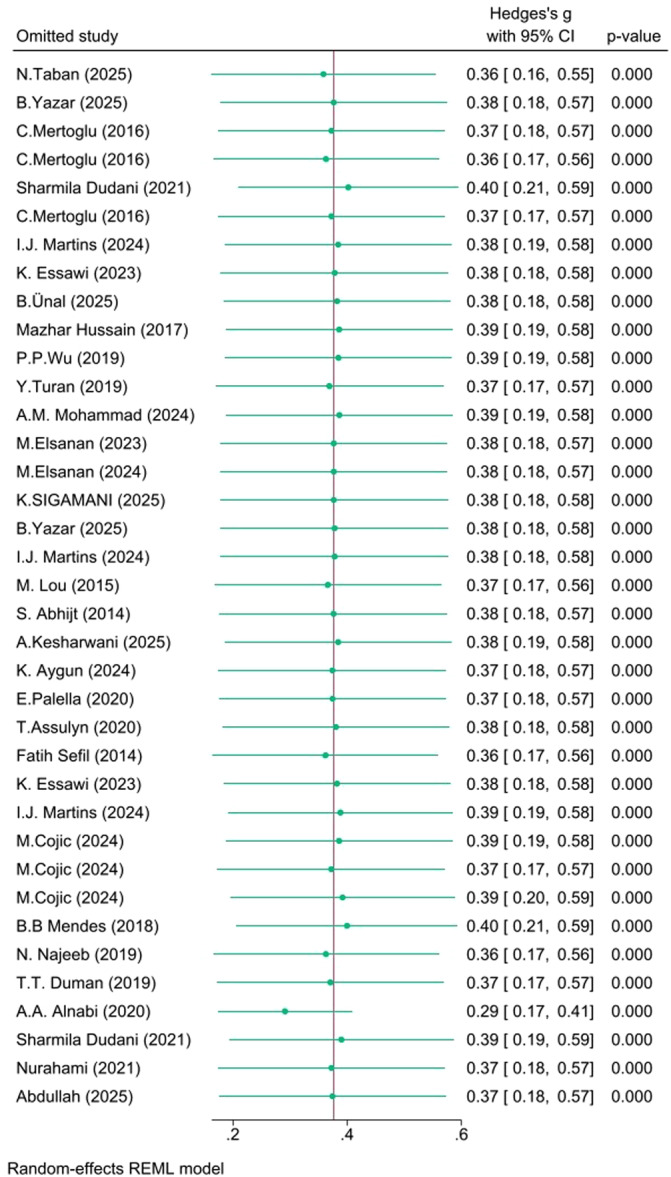
Leave‐One‐out sensitivity analysis (Poor vs Controlled**)**.

**Figure 6:**
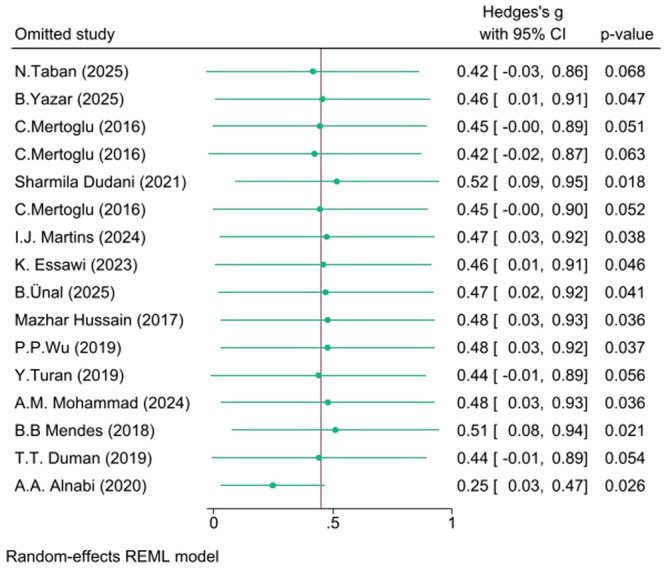
Leave‐one‐out sensitivity analysis (Poor vs Normal).

**Figure 7:**
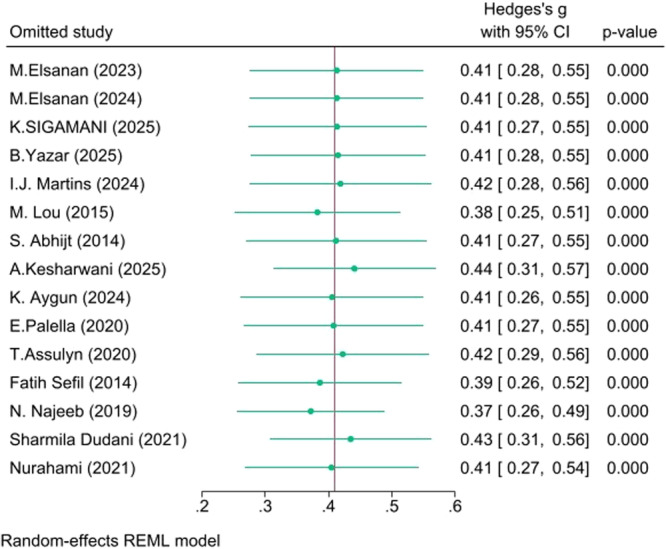
Leave‐one‐out sensitivity analysis (Poor vs Prediabetes).

These sensitivity analyses are detailed in Figures 5, 6, 7.

### 3.4 Risk of Bias Evaluation

The majority of studies were assessed as high or moderate quality based on JBI critical appraisal checklists (Table 2). Low‐quality studies contributed minimally to the overall results, as verified by sensitivity analyses that showed no material change in effect estimates upon their exclusion.

### 3.5 Publication Bias

**Figure 8:**
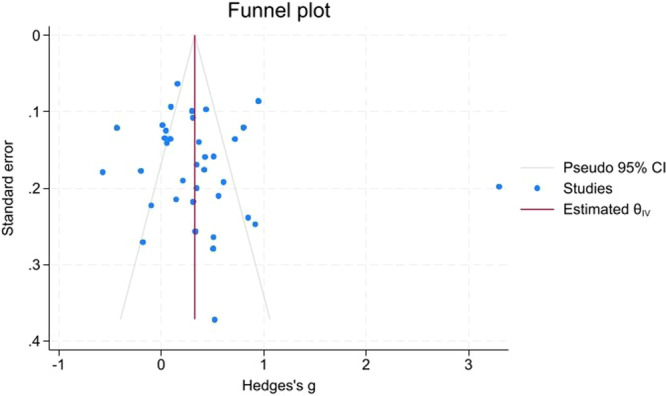
Funnel plot for poor versus well‐controlled T2DM.

**Figure 9:**
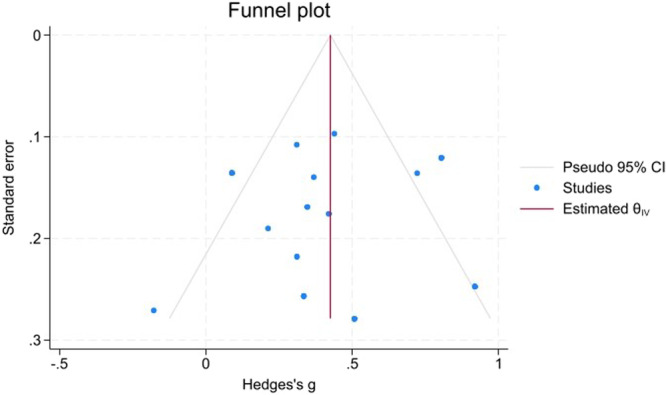
Funnel plot for poor versus prediabetes.

**Figure 10:**
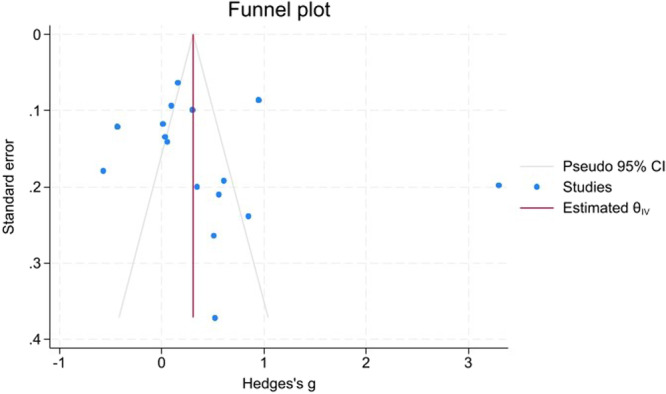
Funnel plot for poor versus normoglycemia.

Funnel plots (Figures 8, 9, 10) demonstrated close symmetry for Poor versus Well‐controlled and Poor versus Normoglycemia comparisons, with minor asymmetry observed for Poor versus Prediabetes.

Publication bias assessment revealed:

- **Trim‐and‐fill analysis**: No imputed studies for Poor versus Well‐controlled or Poor versus Normoglycemia; 3 studies imputed on the right side for Poor versus Prediabetes, suggesting minor positive bias, but the adjusted estimate remained significant (g  =  0.49, 95% CI: 0.35–0.63).
- **Egger's test**: Non‐significant for all comparisons (Poor vs. Well: *p* = 0.456; Poor vs. Prediabetes: *p* = 0.527; Poor vs. Normoglycemia: *p* = 0.350), indicating no small‐study effects.
- **Begg's test**: Non‐significant for Poor versus Well (*p* = 0.200) and Poor versus Prediabetes (*p* = 0.804); approached significance for Poor versus Normoglycemia (*p* = 0.096), suggesting marginal bias that did not materially affect conclusions.

Overall, no substantial publication bias was detected, and the primary findings remain robust after bias adjustment (Table 4).

**Table 4:** Summary of publication bias assessments.

Publication Bias Assessment	Comparison	Parameter/Test	Number	SE/Additional Info	*z*‐value	*p* value	Hedges's *g* (Observed)	95% CI (Observed)	Hedges's *g* (Observed + Imputed)	95% CI (Observed + Imputed)	Interpretation
Trim‐and‐Fill Analysis	Poor vs Well‐controlled	Observed Studies	37	,	,	,	0.376	0.183–0.569	0.376	0.183–0.569	No imputed studies; no publication bias detected
	Poor vs Prediabetes	Observed Studies	15	,	,	,	0.410	0.279–0.540	0.487	0.347–0.626	3 imputed studies (right); minor positive bias possible
	Poor vs Normoglycemia	Observed Studies	16	,	,	,	0.449	0.028–0.871	0.449	0.028–0.871	No imputed studies; no publication bias detected
Egger's Test	Poor vs Well‐controlled	Beta₁ Coefficient	1.15	1.544	0.75	0.4555	,	,	,	,	Not significant; no small‐study effects
	Poor vs Prediabetes	Beta₁ Coefficient	−0.74	1.167	−0.63	0.5265	,	,	,	,	Not significant; no small‐study effects
	Poor vs Normoglycemia	Beta₁ Coefficient	2.68	2.868	0.93	0.3499	,	,	,	,	Not significant; no small‐study effects
Begg's Test	Poor vs Well‐controlled	Kendall's Score	99.00	76.446	1.28	0.1999	,	,	,	,	Not significant; no small‐study effects
	Poor vs Prediabetes	Kendall's Score	−6.00	20.158	−0.35	0.8041	,	,	,	,	Not significant; no small‐study effects
	Poor vs Normoglycemia	Kendall's Score	38.00	22.211	1.67	0.0957	,	,	,	,	Approaches significance (*p* = 0.096); marginal bias

### 3.6 Subgroup Analysis

Table 5 contains the results of subgroup analyses by region of study for each of the three glycemic contrasts evaluated. The combined sample of studies conducted in Asia exhibited larger pooled effect sizes (*g*‐range = 0.43 to 0.54) than North American studies, which typically had smaller or negligible pooled effect sizes (*g*‐range = −0.16 to +0.31). The overall level of heterogeneity was trending to remain substantial in almost all regions (e.g., *I*² = 94.05% for poor vs. well‐controlled; 97.69% for poor vs. “normoglycemic”; *p* > 0.12 for tests of group differences on poor vs. well‐controlled; poor vs. prediabetes, respectively), suggesting that geographical location accounts for only a small percentage of the observed variability across studies.

**Table 5:** Summary of regional subgroup analysis.

Comparison	Overall *k*	Overall g	Overall *I²* (%)	Subgroup with Highest Effect	Subgroup with Lowest Effect
Poor versus Well‐Controlled	37	0.38	94.05	Asia (*g* = 0.47)	N. America (*g* = −0.02)
Poor versus Prediabetes	15	0.41	57.84	Asia (*g* = 0.43)	N. America (*g* = 0.31)
Poor versus Normoglycemia	16	0.45	97.69	Asia (*g* = 0.54)	N. America (*g* = −0.16)

## 4 Discussion

This systematic review and meta‐analysis provide compelling evidence of a significant association between elevated NLR and poorer glycemic control in T2DM, as demonstrated by progressively higher Hedges’ g estimates across worsening glycemic categories. The gradual increase in NLR from normoglycemia to prediabetes and poorly controlled T2DM (*g* ≈ 0.45 → 0.41 → 0.38) supports the concept that NLR reflects the systemic inflammatory burden that accompanies metabolic dysregulation in diabetes. Chronic hyperglycemia creates a pro‐inflammatory milieu that promotes insulin resistance, endothelial dysfunction, and vascular complications, and the higher NLR observed in individuals with poor glycemic control is consistent with this pathophysiological framework [15, 16, 17]. These findings are in line with prior work, including the recent meta‐analysis by Adane et al., which reported a significant relationship between NLR and HbA1c and proposed NLR as a surrogate marker of glycemic control [18]. Similar conclusions were reached by Mahankali et al., Dayama et al., and Duman et al., who all demonstrated higher NLR values in uncontrolled T2DM and positive correlations between NLR and HbA1c, reinforcing the role of NLR as a marker of subclinical inflammation in this population [19, 20, 21].

The bidirectional interplay between T2DM and inflammation is now understood as a complex disturbance of the immune microenvironment and metabolic signaling pathways rather than a simple linear cascade [21, 22]. Chronic hyperglycemia induces oxidative stress and upregulates pro‐inflammatory cytokines such as IL‐6 and TNF‐α, which in turn modulate key signaling hubs including mTOR and related nutrient‐sensing pathways, thereby linking metabolic overload to immune activation and tissue damage [22, 23, 24]. Emerging data also implicate coagulation and fibrinogen pathways, inflammatory genomic signatures, and gut‐microbiome–driven immune activation in the development of diabetic complications, particularly nephropathy and other microvascular sequelae [23, 25, 26]. Findings from cancer and tumor‐microenvironment research have further highlighted how systemic low‐grade inflammation, immune cell trafficking, and stromal remodeling can be orchestrated through networks that overlap with those observed in metabolic disease [16, 27, 28, 29]. Integrating NLR into this broader immunometabolic context supports its interpretation as a readily obtainable composite index of neutrophil‐driven innate activation and relative lymphopenia, capturing aspects of the disturbed immune microenvironment that are not directly reflected by glycemic indices alone [6, 7, 8, 9, 30].

In the present analysis, the poor versus prediabetes comparison demonstrated the most consistent effect (*I*² = 57.84%), with substantially lower heterogeneity than the poor versus well‐controlled and poor versus normoglycemia contrasts. Subgroup analyses by geographic region, summarized in Table 5, showed that Asian studies generally reported the largest effect sizes, whereas North American studies tended to show smaller or null effects. Nevertheless, tests of group differences were not statistically significant for the poor versus well‐controlled and poor versus prediabetes comparisons, and heterogeneity remained high within most strata, indicating that region alone does not fully explain the observed variability. Similarly, stratification by study design did not materially reduce heterogeneity. These patterns suggest that between‐study differences in the magnitude, but not the direction, of the association are substantial and likely reflect unmeasured or inconsistently reported clinical factors rather than fundamental biological differences.

Not all individual studies have demonstrated a strong relationship between NLR and glycemic control. For example, Umarani et al. reported no significant association between NLR and the severity of glucose intolerance [31]. Such discrepancies may arise from differences in sample characteristics (including age and BMI distributions), duration of diabetes, burden of co‐existing inflammatory or cardiovascular conditions, and background treatment regimens. NLR is an inherently non‐specific marker that is highly sensitive to acute and chronic infections, physiological stress, smoking status, and the presence of comorbidities such as coronary artery disease or chronic kidney disease, as well as to medications with anti‐inflammatory properties (e.g., metformin, SGLT2 inhibitors, statins, and certain antihypertensive agents) [23, 30, 32]. Most of the included primary studies relied on crude or minimally adjusted analyses and did not systematically control for these potential confounders. Consequently, part of the observed association between higher NLR and poorer glycemic control may reflect a clustering of inflammatory comorbidities and treatment differences among patients with suboptimal metabolic control, and the findings should be interpreted as associative rather than causal.

Despite these limitations, the clinical relevance of the present findings is considerable. HbA1c remains the gold standard for assessing long‐term glycemic control and for guiding therapeutic decisions [33, 34], but it does not capture the inflammatory processes that underpin many diabetes‐related complications [32, 35]. By contrast, NLR integrates information on innate and adaptive immune cell dynamics and has been linked to cardiovascular and microvascular risk in a range of clinical settings [4, 8, 35, 36]. In this context, NLR should be viewed as a complementary rather than a competing marker, potentially helping identify T2DM patients with an elevated inflammatory burden who are at higher risk of adverse outcomes. A key question for clinical translation is whether a practical NLR cut‐off can be defined. Because the included studies used heterogeneous thresholds, analytic strategies, and populations, and many treated NLR as a continuous variable, it was not possible to derive a robust, unified diagnostic cut‐off in this meta‐analysis. At present, the most defensible use of NLR may be as a relative indicator, either in comparison with contemporaneous controls or through longitudinal self‐comparison within individuals, rather than as a single universal threshold. Furthermore, while NLR is inexpensive and ubiquitously available, it is less specific than markers such as high‐sensitivity CRP or IL‐6 and is more easily influenced by transient conditions; this trade‐off between accessibility and specificity should be acknowledged when considering how NLR might be integrated into risk‐stratification or monitoring algorithms [3, 6].

The robustness of the pooled estimates for the poor versus well‐controlled and poor versus prediabetes contrasts was supported by leave‐one‐out sensitivity analyses, which showed that no single study disproportionately drove the results. In contrast, the poor versus normoglycemia comparison was more sensitive to the exclusion of certain influential studies and therefore warrants more cautious interpretation. Formal assessments of publication bias using Begg's and Egger's tests, supplemented by trim‐and‐fill procedures and funnel‐plot inspection, did not reveal strong evidence of small‐study effects. However, these statistical tests have limited power when the number of available studies is modest, and minor asymmetry, particularly in the prediabetes comparison, where a few studies were imputed on the right side of the funnel plot, suggests that a degree of positive bias cannot be entirely excluded. Accordingly, statements regarding the absence of publication bias should be interpreted with appropriate caution.

Limitations of this meta‐analysis must be acknowledged. First, key clinical variables such as duration of diabetes, smoking status, detailed medication profiles, and the presence of acute infections or inflammatory comorbidities were either inconsistently reported or not reported at all, preventing more refined meta‐regression analyses that could have clarified sources of heterogeneity. Second, although subgroup analyses by region and study design were conducted, heterogeneity remained substantial in most strata, indicating that important effect modifiers remain unaccounted for.

Future research should focus on longitudinal and interventional designs to clarify whether NLR independently predicts incident complications and whether targeted anti‐inflammatory or metabolic therapies can modify both NLR and clinical outcomes. Detailed characterization of the immune microenvironment in T2DM, integrating NLR with fibrinogen and other coagulation markers, cytokine panels, genomic and transcriptomic signatures, and microbiome profiles, may help delineate mechanistic pathways linking systemic inflammation to specific complications such as diabetic nephropathy, retinopathy, and neuropathy [9, 22, 24]. The impact of commonly used anti‐diabetic and cardiometabolic medications, including metformin, SGLT2 inhibitors, GLP‐1 receptor agonists, and statins, on NLR trajectories warrants systematic evaluation, as these agents may exert independent anti‐inflammatory effects. Combining NLR with established inflammatory biomarkers (e.g., CRP, IL‐6, or adiponectin) in multivariable risk models and validating such models across diverse ethnic groups and healthcare settings could enhance risk stratification for adverse outcomes. Ultimately, individual patient data meta‐analyses and well‐designed prospective cohorts will be essential to define clinically meaningful NLR thresholds, characterize dynamic changes over time, and determine whether modulation of NLR translates into tangible reductions in diabetes‐related morbidity and mortality.

## 5 Conclusion

This updated meta‐analysis reinforces a strong and consistent association between elevated NLR and poor glycemic control in patients with T2DM. The observed stepwise gradient in NLR across glycemic categories, together with its established links to cardiovascular and microvascular risk, supports NLR's potential as a simple, inexpensive, and widely available marker of systemic inflammation that can complement, rather than replace, traditional glycemic indicators such as HbA1c. Given the observational nature of the underlying evidence, the non‐specificity of NLR, and the residual heterogeneity between studies, these findings should be interpreted as demonstrating association rather than causation. Longitudinal and interventional studies, particularly those integrating detailed immunometabolic profiling and standardized assessment of confounders, are needed to determine whether NLR can be used to define clinically actionable thresholds, guide individualized risk stratification, and inform therapeutic strategies to reduce the burden of T2DM complications.

## Author Contributions

**Maryam Mohammadi, Parsa Panahiyan, and Faizan Bashir:** leading the conceptualization, literature search, data extraction, analysis, and drafting of the manuscript. **Shayesteh Haghighi, Sara Ahmadi, Ensieh Olama, Yasaman Ghodsi Boushehri, and Elnaz Olama:** assisted with data curation, methodology, and manuscript editing. **Alaleh Alizadeh, Zahra Sadat Hoseini Nasab, and Mahdiyeh Naziri:** contributed to critical revision and interpretation of findings. **Niloofar Deravi, Neda Fakhrghasemi, and Danyal Yarahmadi:** served as co‐corresponding authors, overseeing project administration and approving the final manuscript.

## Funding

The authors received no specific funding for this work.

## Ethics Statement

Not applicable. This is a systematic review and meta‐analysis of previously published data and does not involve human participants, human data, or human tissue.

## Consent

Not applicable. This manuscript does not contain any individual person's data in any form.

## Conflicts of Interest

The authors declare no conflicts of interest.

## Transparency Statement

The lead author Niloofar Deravi, Neda Fakhrghasemi, Danyal Yarahmadi affirms that this manuscript is an honest, accurate, and transparent account of the study being reported; that no important aspects of the study have been omitted; and that any discrepancies from the study as planned (and, if relevant, registered) have been explained.

## Data Availability

Data sharing is not applicable to this article as no datasets were generated or analysed during the current study. All data used were from published articles.
